# Physiotherapeutic Approach in Enhancing Recovery and Quality of Life After Vaginal Hysterectomy: A Case Report

**DOI:** 10.7759/cureus.56057

**Published:** 2024-03-12

**Authors:** Vaishnavi R Waghe, Vrushali Athawale

**Affiliations:** 1 Physical Medicine and Rehabilitation, Ravi Nair Physiotherapy College, Datta Meghe Institute of Higher Education and Research, Wardha, IND; 2 Oncology Physiotherapy, Ravi Nair Physiotherapy College, Datta Meghe Institute of Higher Education and Research, Wardha, IND

**Keywords:** cystocele, rehabilitation, physiotherapy, hysterectomy, uterine prolapse

## Abstract

Uterine prolapse is a manifestation of pelvic organ prolapse distinguished by the descent of the uterus from its normal anatomical position into the vaginal canal. Vaginal hysterectomy is a surgical intervention performed to excise the uterus via the vaginal canal. Hysterectomy is correlated with various complications; thus, prompt mobilization and engagement in physiotherapy are imperative postoperatively. This is a case report of a 78-year-old female who reported a persistent sensation of something protruding from her vagina over the past two years. Investigations revealed a third-degree uterocervical descent, leading to the decision for a vaginal hysterectomy. Commencing on Day 5 post-surgery, early mobilization and a comprehensive physiotherapeutic regimen were implemented, encompassing breathing exercises, upper limb mobility exercises, core strengthening routines, pelvic floor exercises, and postural correction. Evaluation using the Modified Oxford Pelvic Floor Muscle Contraction Scale, Pelvic Floor Impact Questionnaire (PFIQ), and World Health Organization Quality of Life (WHO-QOL) demonstrated notable improvement. The findings suggest that promoting early mobilization and facilitating the rehabilitation of pelvic musculature, along with core strengthening through physiotherapy, plays a pivotal role in expediting recovery and enhancing the overall quality of life for hysterectomy patients, potentially alleviating difficulties in performing daily activities.

## Introduction

Uterine prolapse represents a form of genital prolapse characterized by the descent of the uterus from its normal anatomical position into the vaginal canal [1]. The prevalence of prolapse, marked by a weakening of vaginal or uterine support, varies among women at different stages, ranging from 24% for stage 0, 38% for stage I, to 35% for stage II. Additionally, within the general population, 2-6% of individuals experience total prolapse extending beyond the vaginal entrance, categorized as stage III [2]. A rectocele refers to the protrusion of the anterior rectal wall, typically directed towards the vaginal cavity. Conversely, an enterocele is characterized by the herniation of a peritoneum-lined sac between the vagina and rectum, containing abdominal contents, often including the small bowel [3]. A cystocele is defined as the descent of the anterior vagina, wherein the urethra-vesical junction (located 3 cm proximal to the external urinary meatus) or any anterior point proximal to this, descends to a level less than 3 cm above the plane of the hymen [4].

Multiparity, vaginal delivery, advanced age, and elevated body mass index (BMI) have emerged as prevalent risk factors for prolapse. Vaginal childbirth is notably the primary predisposing factor for the subsequent development of pelvic organ prolapse (POP) and stress incontinence in women under the age of 60 [5,6]. Complications arising from uterine prolapse during pregnancy encompass urinary tract infection, acute urinary retention, and, in severe instances, maternal mortality. In the intrapartum setting, pelvic organ prolapse poses challenges such as inadequate cervical dilatation, increased occurrence of cervical lacerations, and obstructive labor. Early 20th-century observations underscored puerperal infection as a significant repercussion of POP, with prematurity emerging as the principal cause of fetal demise and infection serving as the primary contributor to maternal mortality linked with POP [7].

A comprehensive hysterectomy involves the excision of both the uterus and cervix. The removal of adnexal structures, including the ovaries and fallopian tubes, may or may not be concurrently performed. In a supracervical or subtotal hysterectomy, the cervix is intentionally retained. These surgical interventions are conducted through one of three primary approaches: abdominal hysterectomy (AH), vaginal hysterectomy (VH), or laparoscopic hysterectomy (LH). Risk factors associated with the likelihood of undergoing a hysterectomy include age, BMI, and smoking status. A significant percentage of women report experiencing specific sexual health issues, such as vaginal dryness (31.8%), decreased frequency of orgasm (42.3%), and dyspareunia (37.65%). Notably, an overarching 82.35% of women exhibit sexual dysfunction [8]. Hysterectomy may give rise to post-surgical sequelae, such as post-operative pain at the suture site, generalized weakness, decreased mobility, decreased strength, urinary incontinence, or organ prolapse. These manifestations can be proficiently addressed through timely physiotherapeutic intervention, encompassing Kegel exercises, core strengthening regimens, respiratory exercises, lower limb mobility exercises, bed mobility exercises, and specific yoga poses [9]. Early implementation of physiotherapeutic intervention following gynecological surgery yields significant enhancements in the quality of life for patients [10].

## Case presentation

A 78-year-old gravida 3, para 3, and living 3 (P3L3) female presented with a two-year history of vaginal protrusion, following menopause two decades prior. Initially, she reported discomfort in the pubic region during daily activities, urinary incontinence, a burning sensation during micturition, and abdominal pain. Over the course of six to eight months, these symptoms progressed to the perception of a protruding mass from the vagina. Notably, there was no reported history of bloody discharge. She initially sought care at a local private hospital where temporary relief was achieved with medications and investigations. However, symptoms progressed, leading her to seek evaluation at Acharya Vinoba Bhave Rural Hospital. Abdominal and pelvic ultrasound examinations revealed a diagnosis of third-degree uterocervical descent, accompanied by dryness in the prolapsed area. Additionally, third-degree cystocele, and grade two enterocele and rectocele were identified upon thorough examination. Consequently, the patient underwent a vaginal hysterectomy with sacrocolpopexy to address the pelvic organ prolapse. Postoperatively, the patient reported generalized weakness. Assessment on the Numerical Pain Rating Scale (NPRS) indicated a pain score of 7/10 during movement and 3/10 at rest at the suture site on postoperative Day 2. Pain was mitigated with rest and prescribed medications, although exacerbation occurred with movement, without diurnal variations in symptomatology noted. Continued monitoring and management are imperative to ensure optimal recovery and alleviate postoperative discomfort.

Clinical findings

Following the acquisition of informed consent, a thorough patient assessment was undertaken. In concordance with the provided medical history, the patient articulated a two-year history of vaginal protrusion, which exhibited a progressive exacerbation over the past month. Subsequently, a vaginal hysterectomy was performed. Postoperatively, the patient reported pain in the lower abdomen, particularly intensified during activities involving stair ascent and descent, impeding the ability to bend down and sit on the floor. Furthermore, the patient manifested complaints of constipation and incontinence. The examination performed with the patient in a supine position revealed diminished muscle strength in the core musculature. Local examination exhibited Grade II tenderness at the suture site.

Therapeutic interventions

**Figure 1:**
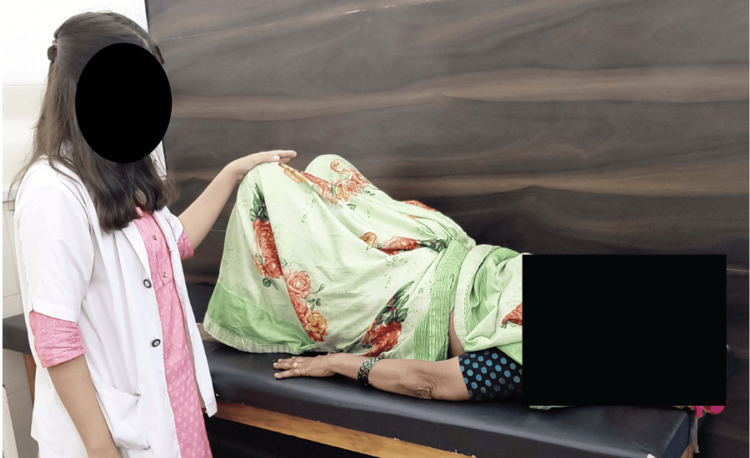
Patient performing pelvic bridging exercise

**Figure 2:**
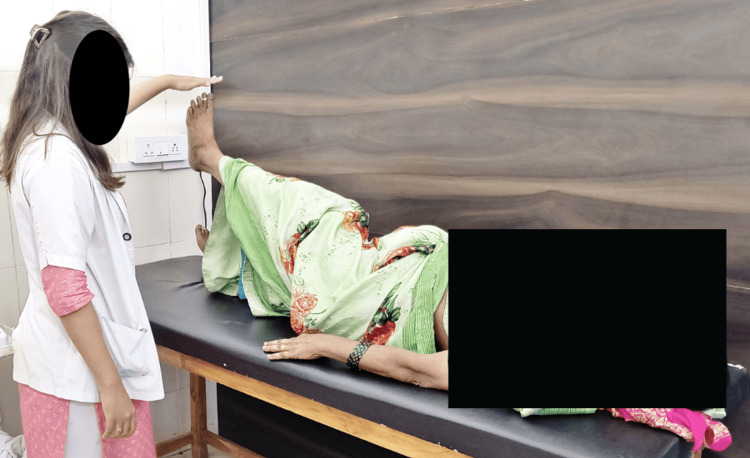
Patient performing straight leg raise

The physiotherapy rehabilitation protocol given for four weeks is shown in Table 1, whereas the exercises performed by the patient are shown in Figure 1 and Figure 2.

**Table 1:** Therapeutic rehabilitation

Goals	Intervention (1-4 weeks)	Repetition
To educate about the post-operative precautions, complications and importance of physiotherapy	Patient education	-
To prevent hospital-acquired diseases, to maintain functional capacity	Breathing exercises Pursed lip breathing Thoracic expansion exercise with upper limb mobility with 5-sec hold	10 repetitions, 1 set
To increase pelvic floor strength	Pelvic floor muscle exercises Kegel’s exercises in supine or sitting position Pelvic bridging exercises	5 repetitions in first week gradually increasing to 8-12 repetitions
To regain core strengthening	Core strengthening exercises for abdominals and back extensors Core isometric contraction Pelvic tilt Cat and camel after 2 weeks Bird dog progression	5 repetitions in first week gradually increasing to 8-12 repetitions
Posture correction	Postural exercises Shoulder shrugs Scapular sets Chin tucks	10 repetitions, 1 set

Follow-up and outcomes

Table 2 shows the pre-treatment and post-treatment outcomes including NPRS, World Health Organization Quality of Life (WHO-QOL), Modified Oxford Pelvic Floor Muscle Contraction Scale, and Pelvic Floor Impact Questionnaire (PFIQ).

**Table 2:** Follow-up and outcomes NPRS: Numerical Pain Rating Scale (7/10: severe pain, 3/10: mild pain) WHO-QOL: World Health Organization Quality of Life scale (39/100: quality of life is moderate, 90/100: quality of life is very good) PFIQ: Pelvic Floor Impact Questionnaire

Outcome Measures	Pre-Treatment	Post-Treatment
NPRS	7/10	3/10
WHO-QOL	39/100	90/100
Modified Oxford Pelvic Floor Muscle Contraction Scale	1/5	5/5
PFIQ	186/300	58/300

## Discussion

In this case, the application of physiotherapeutic techniques, including Kegel exercises, pelvic floor muscle training, and mobilization, demonstrated efficacy in addressing the patient's postoperative challenges. Kegel exercises, designed to aim at pelvic floor musculature strengthening, are effective in preventing and managing urinary incontinence, a prevalent complication subsequent to hysterectomy owing to potential bladder alterations. Furthermore, mobilization exercises encompassing deep breathing maneuvers and progressive ambulation contribute to early recovery and prevent complications such as deep vein thrombosis and respiratory complications. Patient education on proper biomechanics and postural alignment enhances their active engagement in the rehabilitation process and promotes the adoption of health-conscious behaviors. Physiotherapists assume a pivotal role in optimizing functional outcomes and nurturing the holistic well-being of postoperative hysterectomy patients.

The primary goal of physical therapy rehabilitation is to facilitate independence and the resumption of regular, productive activities outside the hospital setting. Darware et al., in their study, observed that administering physiotherapy treatment promptly following gynecological surgery enhances the patient's quality of life. They further noted that a structured exercise program yields greater benefits compared to traditional physiotherapy management. The findings underscore the importance of emphasizing planned exercise regimens for all patients post-gynecological surgeries, highlighting its potential to optimize recovery outcomes and enhance overall well-being [11].

The research conducted by Reddy and Frantz yielded results indicating that conventional modalities for postoperative management of patients subsequent to hysterectomy encompassed mobilization, deep breathing exercises, and educational interventions [12]. As per Fernandes et al., during the postoperative phase subsequent to abdominal surgeries, respiratory physiotherapy has demonstrated efficacy in ameliorating atelectasis and enhancing oxygen saturation levels. In their investigation, Fernandes et al. formulated a respiratory exercise protocol, leading to notable enhancements in minute-volume and tidal volume parameters [13]. Based on prior investigations, hysterectomy has been associated with alterations in bladder function. Kegel exercises are advocated for enhancing pelvic floor muscle strength and mitigating urinary incontinence [14]. Kegel exercises demonstrate a notable capacity to enhance pelvic floor muscle function, medical coping style, and quality of sexual life [15].

## Conclusions

Physiotherapy is integral in promoting optimal recovery and restoring functional capacity in patients following hysterectomy. Physiotherapy interventions through targeted exercises, manual therapy, and patient education, helped to mitigate post-operative complications such as pain and weakness, while also addressing issues like pelvic organ prolapse and incontinence. By fostering early mobilization and facilitating the reconditioning of pelvic musculature, physiotherapy plays a crucial role in expediting the recovery process and improving the overall quality of life for the patient undergoing hysterectomy.
